# The complete chloroplast genome of *Sargassum hemiphyllum* var. *Chinense* (Sargassaceae, Phaeophyceae) and its phylogenetic analysis

**DOI:** 10.1080/23802359.2020.1863165

**Published:** 2021-01-27

**Authors:** Xiaowen Wu, Peng Zhang, Yonghua Zhang, Tiegan Wang

**Affiliations:** a Zhejiang Key Laboratory of Exploitation and Preservation of Coastal Bio-resource, Zhejiang Mariculture Research Institute, Wenzhou, China; b College of Life and Environmental Sciences, Wenzhou University, Wenzhou, China

**Keywords:** *Sargassum hemiphyllum*, chloroplast genome, phylogenomic

## Abstract

*Sargassum hemiphyllum* (Turner) C. Agardh is an important brown macroalga. In this study, we presented the complete chloroplast genome of its variety *S. hemiphyllum* var. *chinense* using genome skimming approach. Circular mapping revealed its sequence length was 124,319 bp, with a large single-copy region (LSC, 73,505 bp) and a small single copy region (SSC, 39,922 bp) separated by a pair of inverted repeats (IRs, 5446 bp). Its chloroplast genome contained 173 genes, including 139 protein-coding, 6 rRNA, and 28 tRNA genes. The phylogenetic analysis indicated that *S. hemiphyllum* var. *chinense* was closely related with *S. confusum*.

*Sargassum hemiphyllum* (Turner) C. Agardh, a kind of brown macroalga that belongs to the Sargassaceae, distributed from Japanese and Korean coasts to the southern Chinese and northern Vietnam coasts (Cheang et al. 2010). As one of the important seaweeds for mariculture, *S. hemiphyllum* is widely applied as food, polysaccharide source, and herbal medicine in China (Cheang et al. 2008). According to previous research, it was divided into two varieties: *S. hemiphyllum* var. *chinense* from China and Vietnam, and *S. hemiphyllum* var. *hemiphyllum* from Japan and Korea (Ajisaka et al. 1997; Cheang et al. 2008). Despite the importance of the species, there has been no genomic studies on *S. hemiphyllum* except the study of its mitochondrial genome (Liu et al. 2016). In this study, we obtained and characterized the complete chloroplast genome of *S. hemiphyllum* var. *chinense* and clarified the phylogenomic relationship with other species in the Phaeophyceae.

One *S. hemiphyllum* var. *chinense* individual was collected from Shenzhen, Guangdong Province of China (N 22°32′52.56″, E 114°34′27.67″), and its specimen was stored at Zhejiang Mariculture Research Institute (Dongtou Base) with an accession number Wu202001. Total DNA was extracted using the modified CTAB method (Doyle and Doyle 1990). Paired-end reads were sequenced on the NovoSeqPE150 platform (Novogene Co., LTD). The draft complete chloroplast genomes were assembled using the program NOVOPlasty (Dierckxsens et al. 2017). The chloroplast genome of *Sargassum horneri* (GenBank accession number: KP881334) was used as the reference sequence. Then, paired-end reads were mapped to the draft genome using CLC Genomics Workbench 11 and yielded the complete chloroplast genome sequence of *S. hemiphyllum* var. *chinense*. Gene annotation was conducted via the online program Dual Organellar Genome Annotator (DOGMA; Wyman et al. 2004) using the same method described by Zhang et al. (2019).

The complete chloroplast genome of *S. hemiphyllum* var. *chinense* (GenBank accession MT800998) is a circular DNA molecule measuring 124,319 bp in length. It comprised a pair of inverted repeat regions (IRs with 5446 bp) divided by two single-copy regions (LSC with 73,505 bp and SSC with 39,922 bp). The nucleotide composition was 34.66% A (43,090 bp), 14.89% C (18,516 bp), 15.68% G (19,493 bp), 34.77% T (43,220 bp). The cp genome encoded a total of 173 genes, including 139 protein-coding, 6 rRNA, and 28 tRNA genes.

All 6 Sargassaceae species with complete chloroplast genome available in the NCBI were selected to reconstruct the phylogenomic tree. Then, maximum likelihood (ML) analyses were implemented on a data set that included 126 protein-coding genes for 12 taxa in Phaeophyceae using RAxML-HPC BlackBox (8.2.12) on CIPRES (http://www.phylo.org) under the GTR model. The phylogenetic tree showed a good resolution of the species of Sargassaceae and other families in Phaeophyceae, with full support at all the nodes. The phylogenomic result showed that *S. hemiphyllum* var. *chinense* is closely related with *S. confusum* (Figure 1).

**Figure 1.**
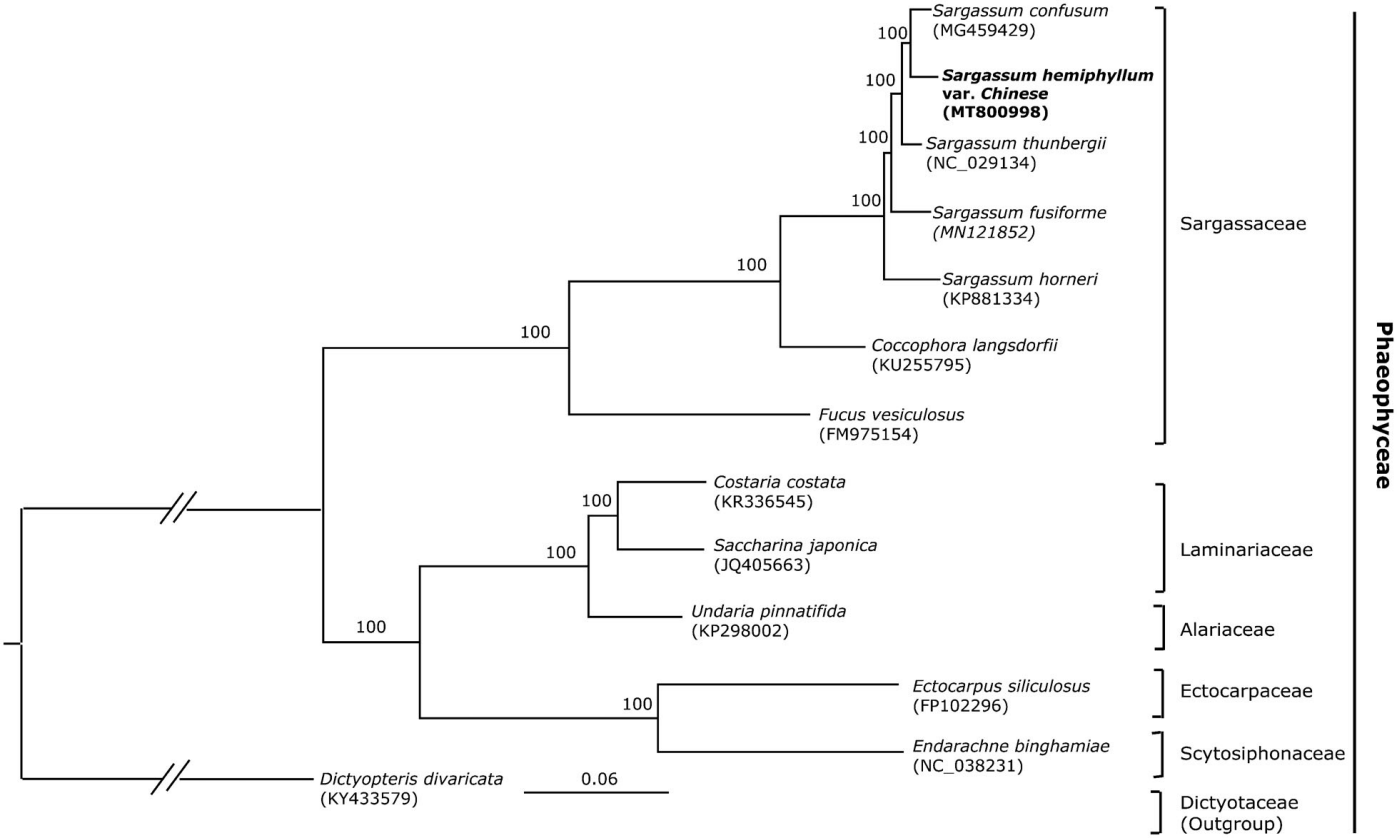
Phylogenetic tree of 13 taxa of Phaeophyceae using ML method. Relative branch lengths are indicated. Numbers near the nodes represent ML bootstrap value.

## Data Availability

The data that support the findings of this study are openly available in Genbank at https://www.ncbi.nlm.nih.gov/genbank/, reference number MT800998. Sequence data of *Sargassum hemiphyllum* var. *chinense* from this article can be found in the NCBI database under BioProject codes PRJNA660488, Sequence Read Archive (SRA) ID SRR12558898.
